# Classification of SINE Tails in the Porcine Genome and Its Potential Impact on *VWA8* Gene

**DOI:** 10.3390/genes17020200

**Published:** 2026-02-07

**Authors:** Yao Zheng, Shasha Shi, Naisu Yang, Chengyu Zhou, Rui Zhou, Hepan Gan, Zhanpeng Gu, Songyu Zuo, Cai Chen, Xiaoyan Wang, Chengyi Song

**Affiliations:** College of Animal Science and Technology, Yangzhou University, Yangzhou 225009, China; mz120180996@yzu.edu.cn (Y.Z.);

**Keywords:** SINEtail-scan, pig, A-rich tail, SINE retrotransposons, *VWA8* gene

## Abstract

**Background/Objectives:** Short Interspersed Nuclear Elements (SINEs) constitute major components of mammalian genomes, but the structural diversity and evolutionary dynamics of their characteristic 3′ poly(A) tails have not been fully characterized. **Methods:** Based on the custom-developed SINEtail-scan pipeline, 1,018,332 SINEs with tail in the pig reference genome (*Sus scrofa* 11.1) were identified and systematically classified, revealing the diversity of tail sequence structures. According to nucleotide composition and microsatellite repeat patterns, the tail sequences were divided into 16 different structural types. **Results:** A-rich sequences predominated (66.3%), while non-A-rich tails exhibited characteristic architectures including AT-format, AC-format, and AG-format repeats. Temporal analysis spanning 85 million years demonstrated progressive tail modification, with A-rich proportions declining from 84.2% in recent insertions to 31.9% in ancient elements, accompanied by accumulation of complex non-A-rich structures. Comparative genomic analysis across 10 pig genome assemblies identified 308 SINE tail insertions within protein-coding sequences, of which 45 (14.6%) exhibited inter-individual structural polymorphism. Detailed investigation of a polymorphic insertion in the *VWA8* gene revealed a 16-bp tail variant causing a frameshift mutation and C-terminal protein structure divergence. **Conclusions:** These findings establish SINE tail sequences as dynamic evolutionary substrates undergoing continuous modification through slippage-mediated mechanisms, with implications for genome evolution, population genetics, and gene function modulation in mammals.

## 1. Introduction

Transposable elements (TEs) constitute a substantial fraction of mammalian genomes, accounting for approximately 45% of the human genome and over 40% of the pig genome [1,2]. Among TEs, Short Interspersed Nuclear Elements (SINEs) represent one of the most successful and widespread families of non-autonomous retrotransposons, which have shaped genome architecture, gene regulation, and species evolution throughout mammalian radiation [3,4]. Unlike autonomous Long Interspersed Nuclear Elements (LINEs), SINEs lack the enzymatic machinery for retrotransposition, and instead rely on LINE-encoded proteins for their mobilization, creating a complex molecular parasitism that has driven co-evolutionary dynamics between these element families [5,6]. SINE elements typically exhibit a tripartite structure consisting of: (I) a 5′ region derived from transfer RNA (tRNA) or 7SL RNA genes that contains an internal RNA polymerase III promoter, (II) a central body region of variable length, and (III) a characteristic 3′ adenine-rich tail at the insertion site [3,7]. While the 5′ and body regions have been extensively studied for their role in transcription and evolutionary relationships, the 3′ tail region has received comparatively less attention despite its critical functional importance. The poly(A) tail serves multiple essential roles in SINE biology: it provides the primer binding site for reverse transcriptase during retrotransposition, contributes to retrotransposition efficiency, and may influence post-insertion element stability and regulation [8,9]. Integration site selection is primarily determined by the sequence preferences of the LINE-1 endonuclease machinery upon which SINEs depend. Recent studies have begun to reveal that SINE 3′ tails are far more structurally diverse than previously appreciated. Rather than consisting of simple homopolymeric adenine tracts, many SINE tails contain complex microsatellite-like repeat structures that arise through a combination of template switching during reverse transcription, replication < 5% in other subfamilies, and post-insertion mutations [10,11]. Roy-Engel demonstrated that Alu element tail lengths exhibit significant variation and correlate with retrotransposition efficiency, with longer A-tails conferring higher mobilization rates [12]. Similarly, Tu provided genomic evidence that slippage retrotransposition drives the relationship between 3′ tandem repeats and poly(dA) tails in mosquito SINEs, demonstrating continuous modification of tail structures over evolutionary time [13]. These observations suggest that SINE tails represent dynamic evolutionary substrates that continue to evolve after genomic integration, potentially generating inter-individual structural variation with functional consequences. The evolutionary dynamics of SINE tail sequences carry important implications for understanding genome evolution and population genetics. Ellegren documented extensive variability in SINE 3′ poly(A) sequences in the pig genome, establishing these regions as abundant genetic markers precisely because of their mutational instability [14]. Borodulina and Kramerov identified distinct classes of mammalian SINEs distinguished by A-rich tail structure in insectivores, supporting the concept that tail architecture serves as a diagnostic feature for SINE classification and subfamily delineation [15]. More recently, Kosushkin demonstrated that retropositional mechanisms of canine SINEs depend critically on A-tail structure, with different tail architectures exhibiting distinct integration preferences and post-insertion stability, highlighting the functional significance of tail sequence variation [16]. Despite these advances, several fundamental questions remain unanswered. First, the complete spectrum of SINE tail structural diversity within a single genome has not been systematically catalogued, limiting our understanding of the evolutionary processes generating tail variation. Second, the temporal dynamics of tail structure modification—how quickly and through what mechanisms simple A-rich tails transition to complex microsatellite structures—remain poorly characterized. Third, the functional consequences of tail structure variation, particularly for SINE insertions within or near protein-coding genes, have been minimally explored. Finally, the extent of inter-individual variation in SINE tail sequences and its potential contribution to phenotypic diversity remain largely unknown. The pig (*S. scrofa*) genome provides an excellent system for addressing these questions. As an important agricultural species and biomedical model organism, the pig genome has been extensively sequenced and annotated, with multiple high-quality chromosome-level assemblies now available from diverse genetic backgrounds [17,18]. SINE elements are abundant in the pig genome, comprising approximately 11% of the total genome content with over one million copies [2]. Previous studies have characterized the major SINE families in pigs (SINEA, SINEB, and SINEC) and established their evolutionary relationships and amplification histories [19]. However, systematic analysis of SINE tail structure diversity and its evolutionary and functional implications has not been performed. Here, we present the first systematic classification and evolutionary analysis of SINE tail sequences across the pig genome, addressing each of these fundamental questions. Using a custom automated pipeline, we systematically analyzed over one million SINE elements, categorizing their 3′ tail sequences into 16 distinct structural types based on nucleotide composition and repeat architecture, thereby establishing the complete structural repertoire of SINE tails. Through temporal analysis spanning 85 million years of evolutionary history, we reveal progressive modification of tail structures from simple A-rich sequences to complex non-A-rich type repeats, elucidating the dynamics and mechanisms underlying tail diversification. Comparative genomic analysis across 10 pig genome assemblies identifies both conserved and polymorphic SINE tail insertions, including 308 insertions within protein-coding sequences that exhibit strong positional and structural constraints, characterizing the functional distribution and selection pressures on SINE tails. Detailed investigation of a polymorphic SINE tail insertion in the *VWA8* gene (von Willebrand factor A domain-containing protein 8) demonstrates direct functional consequences, with a 16-bp insertion variant causing frameshift mutation and altered protein structure, provides computational evidence for inter-individual variation and its potential functional implications, offering candidates for future experimental validation. Our findings establish SINE tail sequences as dynamic evolutionary substrates with implications for genome evolution, population genetics, and gene function in pigs and potentially other mammalian species.

## 2. Materials and Methods

### 2.1. SINE Tail Classification Program Design and Insertion Age Estimation

**Figure 1:**
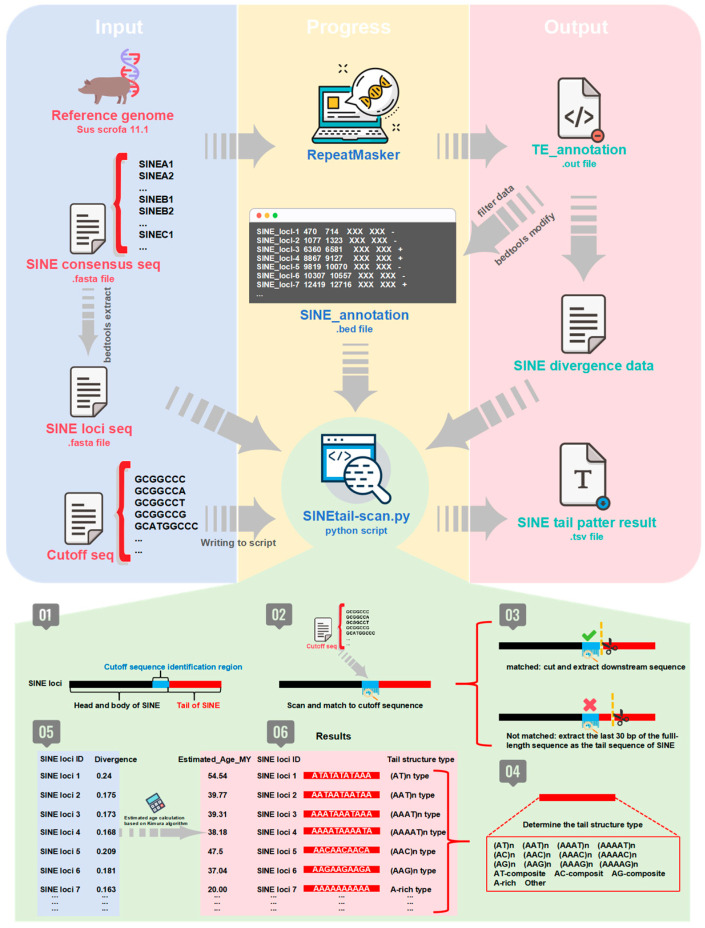
**Schematic overview of the SINEtail-scan pipeline and workflow.** (Top panel) Overall analytical framework. Input data (left, blue panel) comprised the pig reference genome (*S. scrofa* 11.1), SINE subfamily consensus sequences (SINEA1–2, SINEB1–2, SINEC1) in FASTA format, genomic coordinates of SINE insertions extracted via RepeatMasker, and a curated library of cutoff sequences marking SINE body-tail boundaries. Processing steps (middle, yellow panel) involved two sequential stages: (1) RepeatMasker annotation to identify all SINE insertions genome-wide, generating a BED file of SINE coordinates and divergence metrics; (2) custom Python script (SINE_tail_pattern.py) to extract tail sequences and classify structural patterns based on nucleotide composition and repeat architecture. Output files (right, pink panel) included transposable element annotations (.out format), SINE divergence data for age estimation, and tail structure classifications categorizing each insertion by tail type. (Bottom panel) Detailed classification algorithm with six sequential steps. Step 01: Identification of the cutoff sequence demarcating SINE body (black) from tail region (red). Step 02: Pattern-matching algorithm scanning each SINE insertion against the cutoff sequence library. Step 03: Conditional tail extraction—if cutoff sequence matched (top branch), downstream sequence extracted and classified; if unmatched (bottom branch), the terminal 30 bp of full-length sequence designated as tail. Step 04: Tail structure determination via pattern-recognition algorithm identifying nucleotide composition (A-rich, AT-format, AC-format, AG-format) and tail type. Step 05: Integration of tail classifications with divergence calculations and molecular age estimates (million years, MY) for temporal analysis. Step 06: Final categorization into 16 structural types and other rare structural variants.

To characterize structural diversity in SINE 3′ tail sequences, we developed an automated classification pipeline—SINEtail-scan (Figure 1). SINE elements were identified using RepeatMasker (v4.1.2-p1) with a custom library of pig SINE consensus sequences (SINEA1–11, SINEB1–6, SINEC1–8) against the *S. scrofa* 11.1 reference genome. RepeatMasker output was converted to BED format, and individual SINE loci sequences were extracted in FASTA format. Tail boundaries were identified through hierarchical matching of predefined cutoff sequences (Figure S1) that mark the SINE body to tail transition (Figure 1, Steps 01–02). To accommodate post-insertion mutations accumulated during SINE evolution, matching permitted single nucleotide mismatches in addition to exact matches, as individual SINE copies diverge from consensus sequences over evolutionary time. Upon detecting the first match, downstream sequences were extracted as tails. For elements lacking recognizable cutoff motifs, the terminal 30 bp were designated as tails based on sensitivity analysis comparing 20, 30, and 40 bp parameters (Table S1), which demonstrated that 30 bp optimally balances A-rich tail detection sensitivity with microsatellite pattern recognition specificity (Figure 1, Step 03). Extracted tails were classified into 16 categories based on nucleotide composition and repeat features (Figure 1, Step 04). Sequences with ≥70% continuous adenine were classified as A-rich, This threshold was selected based on sensitivity analysis across six threshold values (50–100%), which demonstrated that: (i) the threshold exclusively affects A-rich versus Other category distribution while all structured AN-format categories remain invariant; (ii) 70% provides a biologically meaningful boundary requiring greater than two-thirds adenine content; and (iii) all temporal dynamics and subfamily-specific patterns reported in this study are parameter-independent (Table S1). Non-A-rich tails were analyzed for microsatellite patterns: AT-format [(AAAAT)n, (AAAT)n, (AAT)n, (AT)n, AT-composite], AC-format [(AAAAC)n, (AAAC)n, (AAC)n, (AC)n, AC-composite], and AG-format [(AAAAG)n, (AAAG)n, (AAG)n, (AG)n, AG-composite] (Table 1). Complex architectures were classified as “Other.” For each tail, insertion ages were estimated from RepeatMasker divergence values calculated from complete elements including tails, using the Jukes–Cantor distance model (K = −0.75 × ln [1 − 4D/3], D is proportional divergence) and converted to absolute time using the pig neutral mutation rate of 2.3 × 10^−9^ substitutions per site per year (age = K/[2μ]) [2] (Figure 1, Steps 05–06). The pipeline was embedded in Python 3.8 using Biopython, NumPy (v1.21), and pandas (v1.3). In this study, this calculation assumes constant substitution rates and that observed divergence accumulated neutrally after insertion. Ages were binned into 5-million-year intervals for temporal analysis (0–5 Mya, 5–10 Mya, …, 80–85 Mya). All outputs were compiled in TSV format. The SINEtail-scan integration pipeline has been uploaded to GitHub: https://github.com/zhengyao0804/SINEtail-scan (version 1.0.0).

### 2.2. SINE Identification

SINE elements were identified in the pig reference genome (*S. scrofa* 11.1, assembly GCF_000003025.6) using RepeatMasker (v4.1.2) with SINEA-C consensus sequences (Table S1) [19]. RepeatMasker was executed with the following parameters: -e rmblast -pa 200 -s (sensitive mode) -nolow -no_is (no interspersed repeat small RNA genes) -cutoff 225.

### 2.3. Chromosomal and Functional Element Distribution Analysis

SINE elements with recognizable tail structures (*n* = 1,018,332) were retained for classification analysis, while elements lacking identifiable cutoff motifs or containing highly degraded sequences (*n* = 470,361) were designated as “Other” and excluded from downstream analysis to maintain classification stringency. SINE tail coordinates were intersected with chromosomal locations and functional genomic features using BEDTools v2.30.0 [20]. Chromosomal distribution was assessed by counting SINE occurrences per chromosome and normalizing by chromosome length. Functional element classification utilized Ensembl gene annotation (release 104) defining: protein-coding genes (pcgene), exons, introns, coding sequences (CDS), 5′ untranslated regions (5′UTR), 3′ untranslated regions (3′UTR), and intergenic regions.

### 2.4. Pig Genome

The pig reference genome (*S. scrofa* 11.1, assembly GCF_000003025.6, Duroc) and corresponding gene annotations (Ensembl release 104) were downloaded from Ensembl databases. Nine additional pig genome assemblies representing diverse genetic backgrounds were obtained from NCBI GenBank: Wuzhishan (GCA_048338725.1), Bama (GCA_007644095.1), Meishan (GCA_017957985.1), Ningxiang (GCA_020567905.1), Large White (GCA_044906105.1), Chenghua (GCA_037447515.1), Juema (GCA_040869115.1), Landrace (GCA_963921485.1), and Banna (GCA_041937265.1). All 10 pig genome assemblies used in this study are chromosome-level assemblies with high contiguity (contig N50: 1.0–144.9 Mb, median: 54.5 Mb; scaffold N50: 88.2–144.9 Mb; Supplementary Table S2). Nine assemblies were generated using long-read sequencing technologies (PacBio and/or Oxford Nanopore). These high-quality assemblies ensure reliable SINE tail detection, as contig sizes far exceed SINE element lengths (~300 bp).

### 2.5. Comparative Genomic Analysis Across Pig Genomes

To investigate structural variation in CDS-inserted SINE tails across pig populations, we performed comparative genomic analysis using 10 pig genome assemblies (9 assemblies plus the *S. scrofa* 11.1 reference). Each non-reference assembly was aligned to the reference genome using minimap2 (v2.24) with the asm5 preset optimized for assembly-to-assembly alignment (-cx asm5 -t 80). The resulting PAF alignment files were converted to UCSC chain format using transanno minimap2chain. Reference genome coordinates of 308 CDS-inserted SINE tail loci were lifted over to corresponding positions in each assembly using liftOver, producing assembly-specific BED coordinate files. Tail sequences were extracted from each assembly using bedtools getfasta (v2.30.0) with strand information preserved (-s). For each of the 308 loci, sequences from all 10 genomes were aligned using MAFFT (v7.490) with the L-INS-i algorithm (--localpair --maxiterate 1000) to accurately handle insertions and structural variations. Multiple sequence alignments were manually inspected to identify tail structure polymorphisms, including presence/absence variants, length variations, and sequence composition changes that distinguish different tail categories across individuals.

### 2.6. mRNA Acquisition and Protein Translation

To assess the functional impact of the 16-bp insertion on the VWA8 protein sequence, we reconstructed full-length mRNA transcripts for both the reference Duroc and variant Banna pig genomes. For the Duroc reference genome, the complete *VWA8* mRNA sequence was obtained directly from Ensembl (transcript ENSSSCT00000061114.3). The Banna pig mRNA was reconstructed through a coordinate-guided assembly approach. The orthologous *VWA8* locus in the Banna assembly was first identified using the synteny-based minimap2 alignment described above. All exonic sequences were then extracted by transforming Duroc reference coordinates to their corresponding Banna assembly positions, explicitly accounting for the 16-bp insertion at chr11:25,450,416. The extracted exons were concatenated in proper 5′-to-3′ order, maintaining correct splice junction sequences. Protein translation was performed using the ExPASy Translate tool (https://web.expasy.org/translate/, accessed on 7 November 2025) with the standard vertebrate genetic code [21]. For each mRNA transcript, all six possible reading frames were systematically examined to identify the longest open reading frame (ORF) beginning with a methionine start codon (ATG) and terminating with a stop codon (TAA, TAG, or TGA). Sequence alignment between the Duroc reference and Banna variant proteins was performed using MUSCLE (v3.8.31) to calculate the identity between the two sequences.

### 2.7. Protein Structure Prediction, Analysis, and Visualization

To evaluate the structural consequences of the frameshift mutation on VWA8 protein architecture, we performed comparative three-dimensional structure prediction and analysis. The full-length protein sequences for both Duroc reference (1894 amino acids) and Banna variant (1893 amino acids) were submitted to the AlphaFold3 server (https://alphafoldserver.com, accessed on 8 November 2025) for structure prediction, yielding predicted models in CIF format. Structural alignment between the two protein models was performed using the TM-align web server (https://aideepmed.com/TM-align/, accessed on 8 November 2025), which calculates the TM-score (a metric for assessing topological similarity independent of sequence alignment) and generates superimposed coordinates in PDB format. The similarity value between two proteins was calculated using the online website RCSB (https://www.rcsb.org/alignment, accessed on 8 November 2025). The aligned structures were visualized in tertiary using PyMOL (v2.5), with emphasis on highlighting structural deviations in the C-terminal region affected by the frameshift. Protein domain architecture and secondary structure elements were mapped onto the sequence alignment using ESPript 3.0 (https://espript.ibcp.fr/ESPript/cgi-bin/ESPript.cgi, accessed on 8 November 2025), which generates two-dimensional representations showing the precise correspondence between sequence variations and structural features. This integrated approach enabled comprehensive assessment of how the 16-bp insertion alters protein folding topology and domain organization.

## 3. Results

### 3.1. Structural Diversity of SINE Tail Sequences

To characterize the structural diversity of SINE retrotransposons in the pig reference genome, we systematically analyzed the 3′ tail sequences of all identified SINE elements using the SINEtail-scan program and classified them into 16 distinct structural types based on their sequence composition patterns (Figure 1 and Table 1). These tail types include adenine-rich sequences (A-rich) and various tandem repeat motifs organized into three major categories: AT-format encompasses five subtypes (AAAAT)n, (AAAT)n, (AAT)n, (AT)n, and AT-composite; AC-format includes five subtypes (AAAAC)n, (AAAC)n, (AAC)n, (AC)n, and AC-composite; AG-format comprises five subtypes (AAAAG)n, (AAAG)n, (AAG)n, (AG)n, and AG-composite.

**Figure 2:**
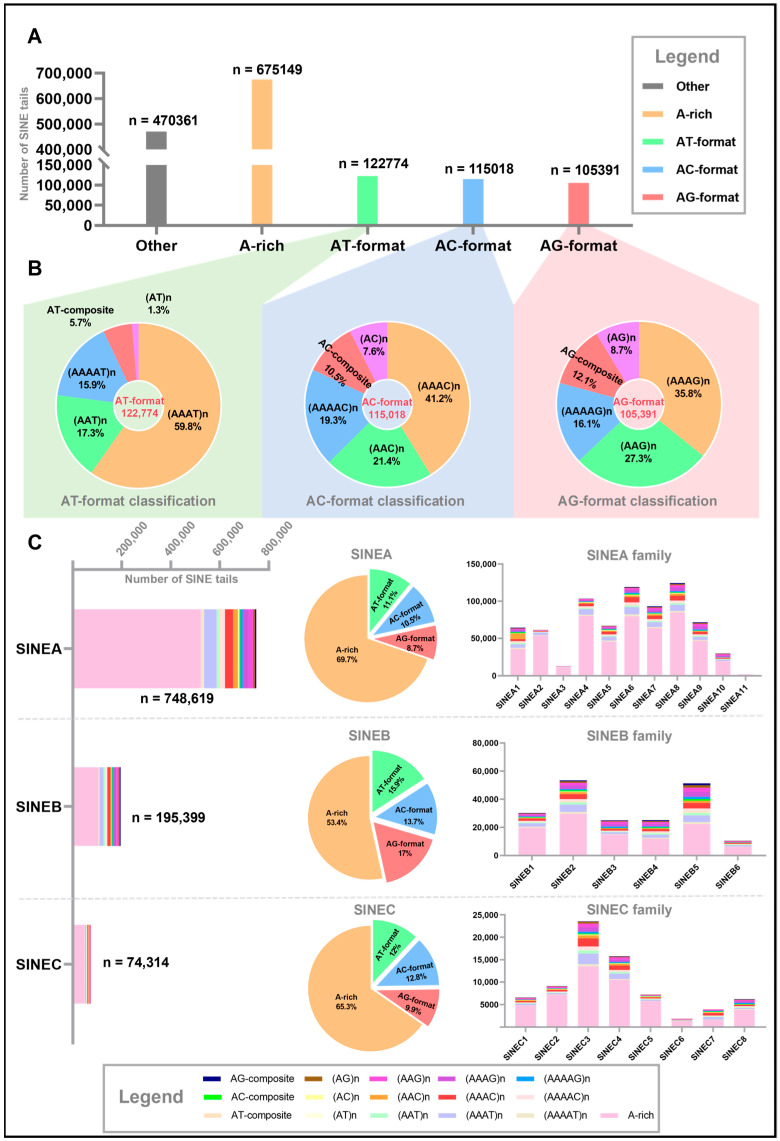
**Systematic classification and distribution patterns of SINE tail structural diversity across the pig genome.** (**A**) Genome-wide distribution of primary tail structure categories identified by the SINEtail-scan pipeline. Bar chart displaying the abundance of four major tail structure types and “Other” among all annotated SINE elements classified using the SINEtail-scan pipeline. (**B**) Proportional composition of subtype classifications within AT-format, AC-format, and AG-format categories. Three pie charts illustrating the relative abundance of detailed subtype classifications within each of the three major non-A-rich type tail formats. (**C**) Family-specific and subfamily-specific distribution patterns of SINE tail structure types. Systematic three-panel analysis of tail architecture variation across three major SINE families (SINEA, SINEB, SINEC) and their constituent 25 subfamilies, organized in three horizontal rows. Each row presents data for one SINE family through three complementary visualizations. Left column: stacked bar charts of 16 tail structure types. These charts display the absolute abundance and compositional makeup of all 16 classified tail types (including A-rich, (AT)n, (AAT)n, (AAAT)n, (AAAAT)n, AT-composite, (AC)n, (AAC)n, (AAAC)n, (AAAAC)n, AC-composite, (AG)n, (AAG)n, (AAAG)n, (AAAAG)n, AG-composite) for each of the three major SINE families. Middle column: pie charts of four major tail categories. These charts present the proportional distribution of the four primary tail structure categories (A-rich, AT-format, AC-format, AG-format) within each SINE family. Right column: subfamily-level stacked bar charts of 16 tail types.

Genome-wide analysis based on the reference genome identified 1,488,693 SINE elements by RepeatMasker, of which 1,018,332 (68.4%) were successfully classified into 16 distinct tail structure types using SINEtail-scan, while 470,361 (31.6%) exhibited unrecognizable or highly degraded tail structures and were categorized as “Other” and excluded from subsequent analysis. Of these 1,018,332 SINE tails, A-rich tail structures predominated, with 675,149 copies (66.3% of all SINE with tail loci) (Figure 2A). The three AN-format categories showed relatively balanced distribution: AT-format contained 122,774 copies (12.1%), AC-format comprised 115,018 copies (11.3%), and AG-format included 105,391 copies (10.3%), collectively accounting for 33.7% of the total SINE with tail population. Within each AN-format category, we observed distinctive hierarchical patterns in tandem repeat structure distribution (Figure 2B, Table S2). Tetranucleotide repeats (AAAX)n consistently emerged as the most abundant subtype in each format, with (AAAT)n representing 59.8% of all AT-format tails, (AAAC)n accounting for 41.2% of AC-format tails, and (AAAG)n comprising 35.7% of AG-format tails. Notably, this pattern reflects a progressive decline in tetranucleotide dominance from AT-format to AG-format, suggesting that tail structure elaboration processes for different repeat motifs are subject to distinct evolutionary pressures or mechanistic constraints. Dinucleotide repeats (AAX)n also showed substantial presence, with (AAT)n, (AAC)n, and (AAG)n each exceeding 20,000 copies and occupying significant proportions within their respective formats. Trinucleotide and mononucleotide repeats, as well as composite structures containing mixed motifs, were present across all three categories at lower abundances, contributing to the overall complexity of SINE tail architecture. This hierarchical organization from longer to shorter repeat units with decreasing abundance may reflect gradual degradation or modification processes of ancestral tetranucleotide structures over evolutionary time. In addition, 470,361 SINE tails (31.6%) were classified as “Other” due to not meeting any of the 16 structural criteria. Characterization of this category revealed a mean length of 24.7 bp (range: 1–43 bp), comparable to classified tails. Nucleotide composition showed elevated but sub-threshold adenine content (52.7% ± 23.8%), with 31.2% of sequences containing 50–70% adenine. Notably, 87.2% retained detectable sub-threshold repeats, indicating that these represent degraded ancestral structures rather than random sequences (Supplementary Table S2). Among the three major SINE families (SINEA, SINEB, and SINEC) (Figure 2C, Table S2), we found SINEA to be most numerous (748,619 copies, 73.5%), followed by SINEB (195,399 copies, 19.2%) and SINEC (74,314 copies, 7.3%). All three families maintained the overall pattern of A-rich dominance, but with varying proportions: 69.7% for SINEA, 53.4% for SINEB, and 65.3% for SINEC. Importantly, non-A-rich types exhibited family-specific characteristics. SINEA showed a slight preference for AT-format (11.1%), higher than AC-format (10.5%) and AG-format (8.7%), while SINEB exhibited a more balanced distribution, with AG-format slightly elevated (17.0%), exceeding AT-format (15.9%) and AC-format (13.7%). SINEC displayed intermediate characteristics, with AT-format (12.0%) and AC-format (12.8%) relatively similar, while AG-format was comparatively lower (9.9%). At the subfamily level, we identified 11 SINEA subfamilies, 6 SINEB subfamilies, and 8 SINEC subfamilies, each exhibiting unique tail structure signatures that can serve as molecular markers for subfamily classification (Figure 2C). Within the SINEA family, SINEA1 was characterized by marked enrichment of (AAC)n repeats (12.2% of total copies, representing 27.2% among non-A-rich tails), distinguishing it from other SINEA subfamilies that typically exhibited (AAAT)n-dominance among non-A-rich structures. SINEA9 and SINEA10 displayed a shift toward AG-type enrichment, with (AAAG)n representing 5.3% of total copies and (AAG)n accounting for 6.1%, indicating unique evolutionary trajectories in tail structure modification that diverge from the family-level AT preference. The SINEB family exhibited more pronounced structural diversification at the subfamily level. Notably, SINEB3 showed exceptional enrichment of (AAAG)n repeats (6.9%) alongside substantial (AAG)n content (6.8%), collectively presenting a strong AG-type signature that aligns with but exceeds the family-level AG enrichment pattern. In contrast, SINEB4 and SINEB5 displayed more balanced distributions across multiple tail types, with no single non-A-rich tail motif exceeding 9.5%, reflecting greater structural heterogeneity that blurs family-level distinctions. Within the SINEC family, SINEC3 stood out with the highest proportion of (AAAT)n repeats (9.9%), while SINEC7 exhibited extremely high levels of both (AAAT)n (14.6%) and (AAAC)n (13.8%), accompanied by the lowest A-rich content (40.4%) among all SINEC subfamilies.

### 3.2. Temporal Dynamics of SINE Amplification and Tail Structure Evolution

**Figure 3:**
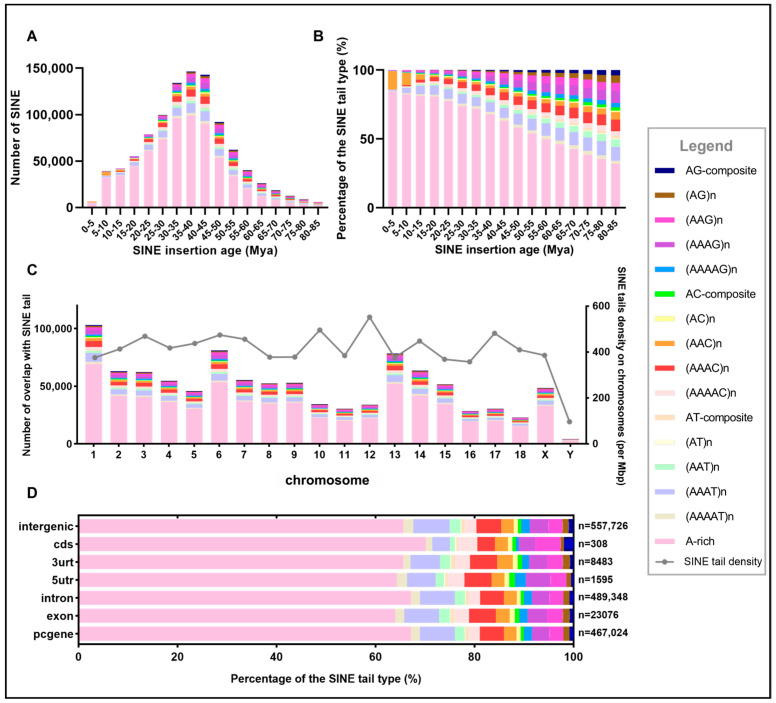
**Temporal dynamics, chromosomal distribution, and functional annotation of SINE tail structural diversity.** (**A**) Temporal distribution of absolute SINE tail abundances across evolutionary time. Stacked bar chart displaying the absolute number of SINE elements categorized by 16 tail structure types across 5-million-year (Mya) time intervals spanning 0–85 Mya. The *x*-axis represents SINE insertion age bins, while the *y*-axis quantifies the total number of SINE elements within each age interval. (**B**) Temporal dynamics of relative SINE tail type proportions. Percentage stacked bar chart illustrating the relative compositional changes of 16 tail structure types across the same 5-Mya time intervals (0–85 Mya). The *x*-axis represents identical age bins as Panel (**A**), while the *y*-axis displays percentage composition normalized to 100% within each temporal bin. (**C**) Chromosomal distribution and density of SINE tail insertions. Dual-axis visualization combining stacked bar charts (left *y*-axis) and line graph (right *y*-axis) to display SINE tail distribution across all pig chromosomes. The *x*-axis represents the 20 pig chromosomes (autosomes 1–18 and sex chromosomes X and Y). Left *y*-axis (stacked bars): absolute number of SINE tail insertions overlapping with each chromosome, partitioned by 16 tail structure types. Right *y*-axis (gray line): SINE tail density calculated as the number of SINE elements per megabase (Mb) of chromosome sequence, providing a length-normalized metric of retrotransposon enrichment. (**D**) Distribution of SINE tail types across genomic functional regions. Horizontal percentage stacked bar chart displaying the compositional profile of 16 tail structure types within seven distinct genomic annotation categories. The *x*-axis represents percentage composition (0–100%), while the *y*-axis lists functional regions ordered by total SINE overlap count (*n* values shown on right).

To investigate the evolutionary dynamics of SINE activity and tail structure modification over time, we estimated insertion ages based on sequence divergence of all SINE with tail copies from subfamily consensus sequences and analyzed tail structure composition across different time periods from 0 to 85 million years ago (Mya) (Figure 3A,B, Table S3). The temporal distribution of SINE insertions revealed a complex amplification history with distinct activity phases (Figure 3A). SINE insertion activity peaked during the 35–40 Mya period, with 146,397 insertions, representing the most intensive period of SINE mobilization in pig genome evolution. This peak was flanked by substantial activity periods, 30–35 Mya (134,056 insertions) and 40–45 Mya (142,804 insertions), with these three periods collectively accounting for approximately 42% of all SINE with tail across all time periods. SINE activity gradually increased from the earliest detectable period (80–85 Mya: 6084 insertions), peaked during the middle Eocene (35–40 Mya), and then progressively declined toward the present. The most recent period (0–5 Mya) contained only 6720 insertions, representing a nearly 22-fold reduction from peak activity levels, indicating significant suppression of SINE retrotransposition in recent evolutionary time. Subfamily-stratified analysis revealed distinct temporal activities among the three SINE families (Figure S2). SINEA represents the youngest and most abundant family (521,327 elements), with peak activity at 30–35 Mya and substantial recent amplification (13.8% of insertions within 0–15 Mya), while SINEB (103,604 elements) and SINEC (48,163 elements) show progressively older distributions, with peaks at 40–45 Mya and minimal recent activity (0.1% and 0.3% in 0–15 Mya, respectively). These temporal profiles demonstrate that the observed tail structure dynamics (Figure 3B) reflect both family-specific amplification histories and age-dependent degradation processes. Analysis of tail structure composition across insertion age categories revealed significant temporal patterns: the proportion of A-rich tails progressively declined with increasing insertion age, while non-A-rich type tails showed the opposite trend (Figure 3B). In the most recent insertions (0–5 Mya), A-rich tails dominated at 84.23%, whereas in the oldest insertions (80–85 Mya), A-rich content decreased to only 31.90%, a reduction of 52.32 percentage points. This negative correlation between insertion age and A-rich content was consistent and monotonic across all examined periods (Figure S3, Table S3). Conversely, the most abundant non-A-rich type in SINE tails—(AAAT)n—showed progressive enrichment in older insertions. In recent insertions (0–5 Mya), (AAAT)n comprised only 0.82%, but this proportion steadily increased to 9.98% in the oldest insertions (80–85 Mya), representing a more than 12-fold increase. Similar age-dependent enrichment patterns were observed for other non-A-rich types, with (AAAG)n increasing from 0.19% to 3.22% and (AAG)n increasing from 0.18% to 5.83%. Notably, (AAC)n repeats exhibited a unique temporal pattern that diverged from other non-A-rich types. In recent insertions (0–5 Mya), (AAC)n showed anomalous enrichment at 13.14%, far exceeding all other non-A-rich types and representing the second-most abundant tail structure after A-rich in this age category. However, this (AAC)n enrichment declined sharply in older insertions, dropping to 8.86% in the 5–10 Mya period, 3.94% in the 10–15 Mya period, and stabilizing at approximately 1–2% in insertions older than 20 Mya. This distinctive temporal signature of (AAC)n distinguishes it from other non-A-rich types, suggesting recent, lineage-specific tail structure modifications. Intermediate age categories (15–50 Mya) exhibited transitional tail structure compositions, with A-rich proportions ranging from 58–81% and progressively increasing diversity in non-A-rich tails. During the peak activity period (35–40 Mya), A-rich tails comprised 67.72% of insertions, with (AAAT)n (6.99%), (AAAC)n (5.11%), and (AAAG)n (3.66%) representing the major non-A-rich type components. This compositional signature remained relatively stable during the major amplification window (30–50 Mya), indicating that SINE retrotransposition during this period primarily generated A-rich structures while maintaining consistent rates of non-A-rich type tail formation.

### 3.3. Chromosomal Distribution

Chromosome-level mapping revealed uneven distribution of SINE elements across the pig genome (Figure 3C). Chr1 harbored the most SINE tail insertions, with 103,061 copies, followed by Chr6 (81,102 copies) and Chr13 (78,335 copies). Smaller chromosomes contained fewer insertions, with Chr18, Chr16, and Chr17 containing 22,958, 28,646, and 30,601 copies, respectively. Although absolute copy numbers positively correlated with chromosome length, normalized density (SINE tails per megabase) revealed substantial inter-chromosomal variation (Figure 3C and Table S4). Chromosome 12 exhibited the highest density (552 per Mb) despite its relatively moderate absolute copy number (34,001), while chromosome 1 and 13, despite harboring the highest absolute copy numbers, displayed comparatively lower densities (both 376 per Mb). A positive correlation between chromosome length and SINE content was observed across all autosomes. Analysis of tail structure composition across chromosomes showed consistent A-rich proportions, ranging from 63.22% (ChrY) to 68.06% (ChrX). Most autosomes clustered between 65–67% A-rich content, including Chr1 (67.09%), Chr6 (65.92%), and Chr13 (66.53%). The distribution of non-A-rich types maintained similar relative proportions across all chromosomes, with (AAAT)n, (AAAC)n, and (AAAG)n consistently representing the most abundant subtypes within their respective AN-format categories.

### 3.4. Functional Element Distribution

To characterize genomic distribution patterns of SINE tails, we mapped all identified SINE tails onto functional genomic annotations (Figure 3D). The analysis revealed relatively balanced distribution of SINE tail insertions between genic and intergenic regions, with 467,024 copies (45.6%) located within protein-coding gene regions (pc-gene) and 557,726 copies (54.4%) in intergenic regions. Within the gene regions, SINE tail insertions showed pronounced bias toward intronic sequences. We identified 489,348 SINE tail copies in introns versus 23,076 in exons, yielding an intron-to-exon ratio of 21.2:1. Analysis of specific gene substructures revealed only 308 SINE insertions in coding sequences (CDS), representing 0.03% of total SINE with tail content. Examination of untranslated regions showed asymmetric distribution, with 8483 insertions in 3′UTRs versus 1595 in 5′UTRs, yielding a 3′UTR-to-5′UTR ratio of 5.3:1. The proportion of A-rich tail structures varied across different functional elements. CDS-associated SINEs exhibited the highest A-rich proportion at 70.13%, followed by introns (67.18%), overall pcgene regions (67.16%), 3′UTRs (65.63%), intergenic regions (65.60%), 5′UTRs (64.26%), and exons (63.95%). Among non-A-rich tail types, (AAAT)n was the most abundant subtype in most functional elements: 7.34% in intergenic regions, 7.07% in exons, 7.04% in introns, 5.94% in 3′UTRs, and 5.83% in 5′UTRs. Notably, the CDS region deviated from this pattern, showing (AAG)n as the most prevalent non-A-rich tail (5.19%), followed by (AAAT)n and (AAAC)n, both at 3.57% (Table S5).

### 3.5. Forty-Five SINE Tail-Mediated Structural Variations in Protein-Coding Gene CDS Regions

To investigate potential functional consequences of rare SINE insertions retained within protein-coding sequences, we systematically analyzed all 308 CDS-associated SINE tail insertions identified in genome-wide annotations. Comparative genomic analysis across 10 chromosome-level assembled pig genomes revealed that 45 of these 308 CDS-inserted loci (14.6%) exhibited structural polymorphism among individuals, with tail structure variations including presence/absence variants, length modifications, and sequence composition changes (Table 2). These 45 structurally polymorphic CDS-inserted SINE tails exhibited several distinctive features. Tail structure analysis revealed overwhelming dominance of A-rich sequences, accounting for 37 insertions (82.2%), while non-A-rich types included (AAAAC)n repeats (2 insertions, 4.4%), AG-composite structures (2 insertions, 4.4%), and single instances of (AAG)n, (AAAC)n, (AC)n, and (AAC)n repeats. Subfamily analysis demonstrated predominance of SINEA family members in structurally polymorphic CDS insertions (40 of 45 cases, 88.9%), with SINEA4 (8 insertions, 17.8%) and SINEA2 (7 insertions, 15.6%) being the most frequent subtypes. SINEB insertions were rare (three cases, 6.7%), and only two SINEC insertions (4.4%) showed structural polymorphism in CDS contexts. Positional analysis within gene structures revealed strong bias toward terminal exons: 23 insertions (51.1%) localized to the last exon, 3 insertions (6.7%) to the second-to-last exon, and 15 insertions (33.3%) to the first exon. Combined, terminal exon positions (first, last, and second-to-last) accounted for 57.8% of polymorphic insertions, while only four insertions (8.9%) occurred in middle exons. This distribution pattern suggests that terminal exon positions provide greater tolerance for SINE insertions, potentially due to reduced disruption of critical protein domains or increased prevalence of non-essential C-terminal extensions. Chromosomal distribution showed notable clustering, with chr13 harboring the highest number of polymorphic insertions (six loci), followed by chr5 (five loci), chr10 (four loci), chr4 (four loci), and chr3 (four loci). The remaining insertions were distributed across chromosomes 1, 2, 6, 7, 8, 9, 11, 12, 15, 17, and 18. This non-uniform distribution may reflect chromosomal variation in insertion tolerance, local recombination rate differences, or population-specific selection pressures. Affected genes spanned diverse functional categories, including structural proteins (KRT12, KRT3), metabolic enzymes (NGLY1, PLA2G7, ADCY7), autophagy regulators (ATG7), E3 ubiquitin ligases (ARIH2), protein phosphatases (PPP4R), membrane trafficking components (RAB8B), extracellular matrix proteins (VWA8, A2M), and signal transduction modulators (NET1, DOCK3), among others.

### 3.6. Impact of SINE Tail Insertion on VWA8 Protein Sequence and Structure

**Figure 4:**
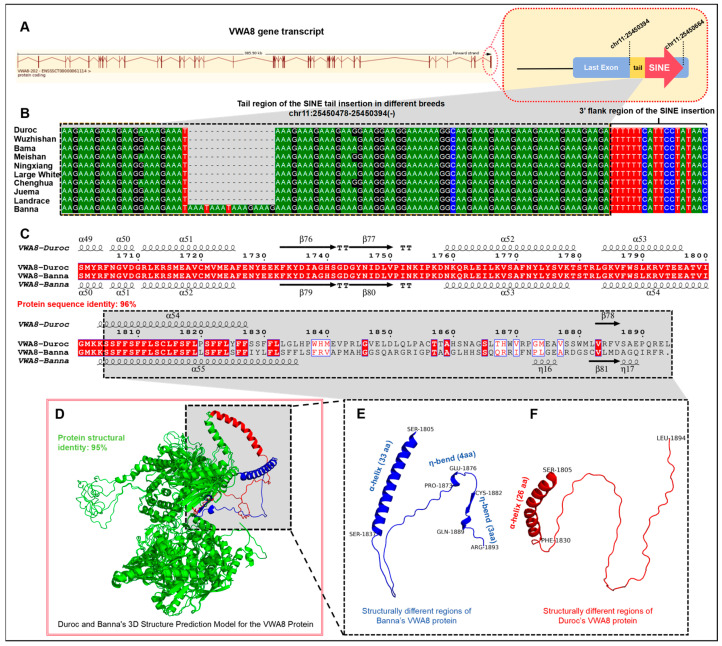
**Functional characterization of a polymorphic SINE tail insertion in the *VWA8* gene and its impact on protein structure.** (**A**) Genomic context and annotation of *VWA8* gene transcript showing SINE tail insertion site. The *VWA8* gene transcript structure from the Ensembl database annotation (transcript ID: ENSSSCG00000001134) is displayed with exon–intron architecture. Red vertical bars represent individual exons. The red dashed box (upper-right inset) highlights the last exon region where a SINE element with tail sequence inserted within the coding sequence at chromosomal coordinates chr11:25450478–25450394(−). This polymorphic insertion occurs within the 3′ terminal portion of the VWA8 coding sequence. (**B**) Multiple sequence alignment of SINE tail region and 3′ flanking sequences across 10 pig breeds. Sequence alignment across 10 assembled pig genomes, with Duroc serving as the reference genome. (**C**) Comparative analysis of VWA8 protein secondary structure between Duroc and Banna individuals. Alignment of predicted secondary structure elements and amino acid sequences for the C-terminal region (amino acids 1701–1893/1894) of VWA8 protein. Secondary structure annotations show α-helix (spiral), β-fold (arrow,), β-turn (TT) and η-bend (spiral). The two protein sequences displayed 96% overall sequence identity. (**D**) Protein tertiary structure comparison of VWA8 between Duroc and Banna breeds. Superimposed tertiary structure prediction models generated using AlphaFold3 algorithms. Three-dimensional protein structure comparison of VWA8 between Duroc and Banna breeds. Superimposed tertiary structure prediction models generated using AlphaFold3 algorithms. Overall structures shown in green (identical/conserved regions) with 95% structural identity across N-terminal and central domains (aa 1–1800). The black dashed box highlights the divergent C-terminal region (aa 1805–1893/1894). Duroc VWA8 C-terminus (red, aa 1805–1894): extended conformation with distinct α-helical content. Banna VWA8 C-terminus (blue, aa 1805–1893): alternative folding topology with different spatial orientation. Overall structures shown in green (identical/conserved regions). The black dashed box highlights the divergent C-terminal region (aa 1805–1893/1894). Duroc VWA8 C-terminus (red, aa 1805–1894): extended conformation with distinct α-helix content. Banna VWA8 C-terminus (blue, aa 1805–1893). (**E**) Detailed protein tertiary structure of Banna VWA8 C-terminal region. High-resolution view of the C-terminal domain (aa 1805–1893, blue). (**F**) Detailed three-dimensional structure of Duroc VWA8 C-terminal region. High-resolution view of the C-terminal domain (aa 1805–1894, red).

To investigate potential consequences of SINE tail insertion within critical genomic regions, we examined a notable case involving the *VWA8* (von Willebrand factor A domain-containing protein 8) gene, where SINE tail sequences were directly inserted into the terminal exon, overlapping with the protein-coding sequence. The SINE retrotransposon in *VWA8* inserted in reverse orientation within the last exon at chr11: 25,450,394–25,450,664. The SINE tail region (chr11:25,450,394–25,450,478) directly overlapped with the protein-coding sequence, with no downstream 3′UTR annotation, confirming integration into a functionally critical region. The insertion was in antisense orientation relative to the *VWA8* gene transcription direction (Figure 4A). To assess the extent of this insertion across individuals and evaluate potential sequence variation within the SINE tail, we examined 10 high-quality chromosome-level pig genome assemblies, including the Duroc reference genome and 9 chromosome-level assembled genomes representing different breeds. This comparative analysis revealed that while the ancestral SINE insertion was universally present across all examined genomes, the SINE tail sequence itself exhibited inter-individual variation (Figure 4B). Sequence alignment of the SINE tail region revealed a 16-bp insertion (-CTTTCTTTATTTATTT- in reference strand orientation; -AAATAAATAAAGAAAG- on the gene-coding strand due to antisense SINE orientation) at position chr11:25450456 relative to the reference genome in one of the examined genomes (Banna pig genome) (Table S6). This 16-bp insertion was absent in the reference genome and the other eight non-reference assemblies, representing a polymorphic site within the SINE tail structure. Translation analysis of the *VWA8* transcript (ENSSSCT00000061114.3) revealed that the presence or absence of the 16-bp insertion had direct consequences for the encoded protein. In genomes lacking the insertion (reference and eight others), *VWA8* gene encoded a protein of 1894 amino acids. In the genome containing the 16-bp insertion, the additional sequence introduced a frameshift mutation beginning at amino acid position 1825, resulting in an altered C-terminal sequence and a protein of 1893 amino acids due to an earlier stop codon. To assess potential structural impacts of the frameshift mutation on VWA8 protein, we analyzed the secondary structure of the two protein variants, and the results showed that their overall sequence identity was 96% (Table S7). Importantly, divergence was strictly confined to the C-terminal region (residues 1805–1894 aa in the reference variant; 1805–1893 aa in the frameshift variant). Notably, the C-terminal regions exhibited distinct prediction confidence profiles: the Banna mini-pig VWA8 (carrying the SINE insertion) showed moderate confidence (Table S8), suggesting structural flexibility, while the Duroc VWA8 (lacking the insertion) displayed low confidence, consistent with intrinsic disorder. These divergent confidence scores suggest that the SINE tail variation may influence C-terminal structural properties. The N-terminal and central regions (residues 1–1804), which contain all VWA8 core domains responsible for the protein’s primary functions in protein–protein interactions and extracellular matrix assembly, remained completely identical between variants (Figure 4C). We performed three-dimensional structure predictions using AlphaFold3 for both protein variants (Figure 4D–F). The predictions indicated that the N-terminal and central regions containing all of the VWA core domains adopted 95% similar structures in both variants, consistent with their sequence identity. This suggests that despite C-terminal alterations, the core functional architecture of the protein is preserved. In contrast, the C-terminal regions displayed distinct predicted conformational features. Sequence–structure alignment showed that structural similarity was maintained through residue 1820, beyond which the frameshift-altered sequence resulted in divergent predicted structures (Figure 4C). The reference protein exhibited a C-terminal region with relatively extended, irregular conformation featuring multiple loop and turn structures. The frameshift variant displayed several distinctive predicted structural features in the altered region: an extended α-helical segment (Ser1805–Ser1837) spanning approximately 33 residues, compared to a shorter, interrupted helical segment in the reference structure. This predicted increase in helical content likely reflects the altered amino acid sequence imposed by the frameshift. Two sharp directional changes (η-bends) at Pro1873–Glu1876 and Cys1882–Gln1889 in the frameshift variant create compact turn motifs not predicted in the reference structure. These features introduce local conformational constraints that may affect surface residue presentation. An overall more compact topology is predicted in the frameshift variant compared to the more loosely organized reference C-terminus (Figure 4C–F). These structural predictions suggest that the 16-bp SINE tail insertion, through introduction of a frameshift at position 1825, may lead to substantial conformational remodeling of the VWA8 C-terminal region while preserving the core VWA domain architecture. The distinct predicted C-terminal structures may have functional implications. The frameshift variant’s compact, helix-rich conformation contrasts with the extended, irregular structure of the reference protein, potentially affecting protein stability, surface accessibility, and interaction interfaces in this region. These conformational differences could influence C-terminal functions such as protein–protein interactions or subcellular localization, though experimental approaches would be necessary to determine phenotypic consequences.

## 4. Discussion

### 4.1. Structural Diversity and Evolutionary Elaboration of SINE Tail Sequences

Our systematic characterization of 1,018,332 SINE elements with tail reveals remarkable structural diversity in tail sequences, organized into 16 distinct categories that reflect both ancestral architecture and lineage-specific modifications. The predominance of A-rich tails (66.3% of all SINEs) represents the canonical structure typical of mammalian SINE elements, consistent with their origin through reverse transcription of RNA polymerase III transcripts terminating at oligo(dT) tracts [5,22]. However, the substantial presence of non-A-rich type tails in the remaining 33.7% indicates that post insertion modifications have elaborated simple A-rich structures into complex repeat arrays [1,23,24]. The hierarchical organization of non-A-rich tails provides insight into molecular mechanisms underlying tail diversification. Roy-Engel demonstrated that SINE tail size variations significantly affect retrotransposition efficiency, with longer A-tails conferring higher mobilization rates [25]. Our observation that tetranucleotide repeats consistently dominate each AN-format category—(AAAT)n at 59.8% in AT-format, (AAAC)n at 41.2% in AC-format, and (AAAG)n at 35.7% in AG-format—suggests that these structures represent stable intermediate states in tail evolution. The progressive decline in tetranucleotide-dominance across formats likely reflects differential stability against spontaneous mutations, with AT-rich sequences particularly prone to slippage-mediated expansions [14,26]. Borodulina and Kramerov identified two distinct classes of mammalian SINEs distinguished by A-rich tail structure in insectivores, supporting the concept that tail architecture serves as a diagnostic feature for SINE classification [15]. Family- and subfamily-specific tail signatures demonstrate that SINE lineages have undergone independent evolutionary trajectories. The observation that SINEA exhibits AT-format preference (11.1%), SINEB shows AG-format enrichment (17.0%), and SINEC displays intermediate characteristics parallels findings in other taxa where SINE families maintain distinct 3′ end structures. Kögler documented divergence of 3′ ends as a primary driver of SINE evolution in Salicaceae, with different families acquiring lineage-specific tail modifications [27]. At the subfamily level, SINEA1’s remarkable enrichment of (AAC)n repeats (27.2% of non-A-rich tails) represents a lineage-specific structural signature comparable to the OsSN SINE families in rice, which evolved novel poly(A) structures at their 3′ ends [28]. The extreme tail remodeling in SINEC7, with only 40.4% A-rich content accompanied by high (AAAT)n and (AAAC)n levels, suggests relaxed functional constraints or distinct insertion site preferences similar to BmSE SINE families in silkworm that acquired (ATTT)n repeat 3′ ends [29].

### 4.2. Temporal Dynamics and Mechanistic Insights into Progressive SINE Tail Modification

The temporal distribution spanning 85 million years reveals complex SINE amplification dynamics, with peak activity during the middle Eocene (35–40 Mya: 146,397 insertions). This peak coincides with the evolutionary radiation of Suina following divergence from other Artiodactyla lineages approximately 50–60 Mya [2,30]. The subsequent 22-fold activity decline reflects progressive inactivation of source genes or increasing efficiency of cellular defense mechanisms, consistent with genome-wide patterns of declining transposable element activity across mammalian evolution [31]. The subfamily-specific temporal profiles (Figure S2) reveal that SINEA elements dominate recent evolutionary periods, while SINEB and SINEC represent progressively older amplification waves, consistent with sequential inactivation of SINE source genes. The progressive tail degradation from A-rich to complex structures observed across evolutionary time (Figure 3B) occurs within individual subfamilies, confirming that these patterns reflect post-insertion modification rather than differential family composition across age bins. The striking inverse correlation between insertion age and A-rich proportion—declining from 84.23% in recent insertions to 31.90% in ancient insertions—demonstrates progressive tail modification over evolutionary time. This pattern reflects multiple molecular mechanisms operating post-insertion. Tu provided genomic evidence that slippage retrotransposition drives the relationship between 3′ tandem repeats and poly(dA) tails in Aedes aegypti SINEs, demonstrating that simple A-rich sequences undergo continuous modification through replication errors [32]. The consistent enrichment of (AAAT)n in older insertions (0.82% to 9.98% across the age spectrum) reflects preferential formation and expansion of this motif within AT-rich substrates. Ellegren documented extensive variability in SINE 3′ poly(A) sequences in the pig genome, establishing these regions as abundant genetic markers precisely because of their mutational instability [33]. The anomalous temporal pattern of (AAC)n repeats—maximum abundance (13.14%) in recent insertions but declining to 1–2% in insertions older than 20 Mya—suggests recent lineage-specific modifications. This pattern could reflect activation of a new SINE subfamily with intrinsic (AAC)n content, as observed in salmon SINEs where novel families can incorporate LINE-related 3′-tails from other SINEs through molecular recombination mechanisms [34]. The subfamily-specific enrichment of (AAC)n in SINEA1 (12.2% of total copies) supports recent amplification with an altered tail structure template. Dewannieux demonstrated that LINE-mediated retrotransposition can mobilize Alu sequences with modified 3′ structures, establishing precedent for tail structure variations arising through retrotransposition machinery interactions [5].

### 4.3. Genomic Distribution Patterns and Selection Constraints

The functional element distribution reveals stringent selection against CDS insertions, evidenced by the dramatic 21.2:1 intron-to-exon ratio and extreme CDS rarity (0.03%). This pattern reflects fundamentally different selection pressures: SINE tail inserts to CDS typically disrupt reading frames or introduce premature termination, imposing severe fitness costs [35]. Chen documented that retrotransposon evolution significantly impacts both lncRNA and protein-coding genes in pigs, with coding sequences experiencing the strongest purifying selection [19]. The preferential 3′UTR accumulation (5.3:1 ratio versus 5′UTR) reflects functional asymmetry, as 5′UTRs contain critical translation initiation elements, while 3′UTRs tolerate insertions more readily [36]. Choi demonstrated that SINE retrotransposons can import polyadenylation signals to 3′UTRs in dogs, suggesting some insertions may acquire regulatory functions in non-coding contexts [37].

### 4.4. SINE Tails Inserted in CDS: Molecular Mechanisms and Evolutionary Constraints

The identification of 308 SINE tail insertions in CDS regions, representing approximately 1 insertion per 3305 total genomic SINEs, underscores strong purifying selection against coding sequence disruption. Comparative genomic analysis across 10 pig genome assemblies revealed that 45 of these 308 CDS-inserted loci (14.6%) exhibit structural polymorphism among individuals, providing a unique window into the evolutionary dynamics of transposable elements within protein-coding contexts. This polymorphism rate substantially exceeds background genomic variation rates, suggesting that CDS-inserted SINE tails either experience accelerated modification through elevated mutational pressure or represent recent insertion events still undergoing selection-driven fixation or elimination.

The retained CDS insertions occupy a highly constrained evolutionary niche defined by multiple selective filters. First, positional tolerance emerges as a critical determinant: 57.8% of polymorphic insertions localized to terminal exons (first, last, or second-to-last), where frameshift disruptions minimally affect protein function or can be tolerated through alternative splicing mechanisms. This pattern mirrors observations in human Alu insertions, where coding sequence insertions predominantly accumulate in terminal exons and are frequently subjected to exonization—the process by which intronic Alu sequences are incorporated into mature mRNA transcripts [38]. The preferential retention of insertions in first exons (33.3%) may reflect 5′ UTR overlap or non-coding leader sequences that buffer against functional disruption. Second, structural simplicity represents a universal feature of CDS-tolerant SINE tails: 82.2% exhibited simple A-rich sequences, while complex microsatellite repeats remained rare. This dominance likely reflects multiple selective pressures, as A-rich tails minimize frameshift lengths and are less likely to form stable secondary structures that could disrupt mRNA processing. Kosushkin demonstrated that retropositional mechanisms of canine SINEs depend critically on A-tail structure, with different tail architectures exhibiting distinct integration preferences and post-insertion stability [16]. Third, subfamily-specific patterns suggest intrinsic structural features modulate CDS compatibility. The SINEA subfamily dominates polymorphic CDS insertions (88.9%), while SINEC insertions remain nearly absent (4.4%), despite SINEC representing 7.3% of total genomic SINEs, indicating fundamental incompatibility with coding sequence constraints [39].

### 4.5. Inter-Individual Variation: The VWA8 Case Study and Population Genetics Implications

The *VWA8* gene exemplifies how SINE tails continue evolving post-integration, generating inter-individual structural variation. Comparative analysis across 10 chromosome-level assemblies revealed universal presence of the ancestral SINE insertion, indicating ancient integration, yet the tail sequence exhibits polymorphism through a 16-bp insertion (AAATAAATAAAGAAAG) at chr11:25450456 in one genome. This demonstrates that even within protein-coding sequences under stringent selection, SINE tails remain substrates for ongoing mutational processes [40]. Mammalian SINE mobilization requires only a 3′ poly(A) tail [41], yet post-insertional evolution generates diverse tail structures. The 16-bp insertion is predicted to introduce a frameshift in the C-terminal region while preserving the N-terminal VWA domains. This observation has important implications: frameshift-inducing insertions can be tolerated when affecting regions under relaxed constraints. The C-terminal region shows evolutionary variability across mammals and lacks conserved functional motifs, suggesting it functions as a flexible linker rather than a structured domain. The molecular mechanism underlying this insertion most likely involves replication slippage during DNA synthesis, facilitated by the AT-rich composition (75% A + T) and simple repeat structure [14]. The presence in only 1 of 10 genomes suggests either recent origin or low-frequency maintenance, possibly reflecting neutral drift without strong selection [42]. From a population genetics perspective, this variant likely falls into the “nearly neutral” category, where selection coefficients are small relative to drift [43]. Zheng reported similar SINE tail variations in the CA5B gene, showing breed-specific patterns associated with production traits [44]. The *VWA8* case extends this observation by demonstrating that tail polymorphisms can occur within CDS regions, although the actual functional consequences require experimental validation. These findings establish that SINE tail polymorphisms represent valuable markers for population genetic studies and potentially trait mapping if functional associations emerge from larger-scale analyses. It is important to note that these structural predictions, while informative, represent computational models requiring experimental validation. Without direct protein-level characterization, several possibilities remain to be resolved: the Banna variant may produce the truncated/altered C-terminal protein as predicted; the transcript containing the frameshift-inducing insertion may be subject to nonsense-mediated decay (NMD) or other RNA surveillance mechanisms, resulting in reduced or absent protein expression; or alternative splicing or translation initiation sites may partially compensate for the frameshift. Experimental approaches such as Western blotting with C-terminal-specific antibodies, mass spectrometry-based protein identification, or RT-PCR coupled with Sanger sequencing of expressed transcripts would be valuable to determine the actual molecular outcomes in Banna pig tissues. Similarly, RNA-seq analysis could reveal whether transcript abundance differs between Banna and Duroc variants, potentially indicating NMD activation. These experimental validations would clarify whether the observed genomic variation translates to protein-level consequences or is buffered through post-transcriptional mechanisms.

## 5. Conclusions

Our systematic analysis reveals a complex evolutionary landscape of SINE tail sequences shaped by initial integration mechanisms, progressive post-insertion modifications, and stringent selection constraints. The identification of 16 structural types among 1,018,332 elements demonstrates remarkable plasticity, with family-specific signatures reflecting independent evolutionary trajectories. Temporal analysis spanning 85 million years reveals progressive modification from A-rich simplicity toward non-A-rich complexity, punctuated by lineage-specific structural diversification such as (AAC)n enrichment in SINEA1. The extreme CDS rarity (0.03%) underscores intense purifying selection, yet the 45 retained insertions demonstrate that specific combinations of positional tolerance, structural simplicity, and functional redundancy permit survival. The *VWA8* case exemplifies continued tail evolution even within CDS regions, generating inter-individual variation with apparently tolerated functional consequences. These findings illuminate transposable element dynamics in mammalian genomes and provide a foundation for investigating functional impacts and population genetics of SINE-mediated structural variation in livestock and other species. Future research should prioritize experimental validation of predicted structural consequences, functional assessment through cellular assays, expanded population sampling, and comparative genomics across species to fully elucidate the evolutionary significance of SINE tail polymorphisms.

## Figures and Tables

**Table 1:** SINE tail structural features classification information.

Type of Tail Structure	Filter Criteria	Characteristic	Example	Quantity
(AAAAC)n	The -AAAAC- structure appears 2 or more times in a row in the tail sequence	-AAAACAAAAC-	-AAAACAAAACAAAACAAAAGACCAAAAA-	23,498
(AAAAG)n	The -AAAAG- structure appears 2 or more times in a row in the tail sequence	-AAAAGAAAAG-	-AAAGAAAAAAGAAAAGGAAAAGAAAAGAA-	17,011
(AAAAT)n	The -AAAAT- structure appears 2 or more times in a row in the tail sequence	-AAAATAAAAT-	-AAAATAAAATAAAATAAAAA-	19,555
(AAAC)n	The -AAAC- structure appears 2 or more times in a row in the tail sequence	-AAACAAAC-	-AAAACAAACAAACAAAAAA-	50,056
(AAAG)n	The -AAAG- structure appears 2 or more times in a row in the tail sequence	-AAAGAAAG-	-AAAAAAAAAAAAAGAAAGAAAGAAAGAAA-	37,681
(AAAT)n	The -AAAT- structure appears 2 or more times in a row in the tail sequence	-AAATAAAT-	-AAATAAATAAATAAATAAATAA-	73,391
(AAC)n	The -AAC- structure appears 2 or more times in a row in the tail sequence	-AACAAC-	-ACAACAACAACAACAACAACAACAACAAAA-	26,064
(AAG)n	The -AAG- structure appears 2 or more times in a row in the tail sequence	-AAGAAG-	-AAAAAAGAAGAAGA-	28,740
(AAT)n	The -AAT- structure appears 2 or more times in a row in the tail sequence	-AATAAT-	-AGGCAAATAATAATAATAATAATAATAAAA-	21,192
(AC)n	The -AC- structure appears 3 or more times in a row in the tail sequence	-ACACAC-	-AACACATACACACACACA-	8998
(AG)n	The -AG- structure appears 3 or more times in a row in the tail sequence	-AGAGAG-	-AAAAAAAAAAGAGAGAGAGAGAAA-	12,792
(AT)n	The -AT- structure appears 3 or more times in a row in the tail sequence	-ATATAT-	-AATATACATTATATATATAAAAGGAAAAAA-	1607
A-rich	-A- base appear in the tail sequence for 5 or more consecutive times and the length of PolyA covers more than 70% of the tail sequence.	-AAAAA-	-AAAAAAAAAAAAAAAAAAAAAA- -TAAAAAGCAAAAAAAAAAAAAAAAAAA-	675,149
AC-composite	Co-occurrence of at least two different AC-series repeat motifs	-AAAAC/AAAC/AAC/AC-	-GTCCTACAAAGCAAAAAACAAACAACAACA-	6402
AG-composite	Co-occurrence of at least two different AG-series repeat motifs	-AAAAG/AAAG/AAG/AG-	-AAAAGAGAAGAAG-	9167
AT-composite	Co-occurrence of at least two different AT-series repeat motifs	-AAAAT/AAAT/AAT/AT-	-AAAAATAAATAATAAATAAAA-	7029
Other	Sequences that did not meet the criteria for composite repeats, single repeats, or A-rich classification	-	-CTCCGACTCAACCCCTAGCCTGGGAACTCC- -TTGGGTGCAGTCCTAAAAA- -TCTGGCTGTGGCTGTGGCTGGCAGCTGCAGTT-	470,361

**Table 2:** Forty-five SINE tail insertions in the CDS region of the protein-coding gene.

SINE Tail_Loci Chromosome:Start-End	Tail Type	SINE Type	SINE Orientation	Gene Name	Transcript ID	Inserted Exon Region *
chr1:108,780,134–108,780,157	A-rich	SscSINEA9	Forward	*RAB8B*	ENSSSCT00000056693.3	Last one
chr1:246,112,991–246,113,005	A-rich	SscSINEA4	Reverse	*NIPSNAP3B*	ENSSSCT00000045553.3	Second-to-last
chr1:89,969,124–89,969,152	A-rich	SscSINEA6	Forward	*ENSSSCG00000004477*	ENSSSCT00000004946.3	Last one
chr2:132,380,965–132,380,987	A-rich	SscSINEA8	Forward	*ENSSSCG00000033503*	ENSSSCT00000055092.2	First one
chr2:81,394,596–81,394,610	A-rich	SscSINEA8	Reverse	*ENSSSCG00000062872*	ENSSSCT00000095214.1	Last one
chr3:107,063,953–107,063,972	A-rich	SscSINEA6	Forward	*ENSSSCG00000036197*	ENSSSCT00000066192.2	Last one
chr3:24,185,633–24,185,663	A-rich	SscSINEA5	Forward	*PDZD9*	ENSSSCT00000027245.4	First one
chr3:43,779,288–43,779,303	A-rich	SscSINEA9	Forward	*NT5DC4*	ENSSSCT00000008865.4	Last one
chr3:79,857,024–79,857,038	A-rich	SscSINEA9	Forward	*COMMD1*	ENSSSCT00000081606.1	Last one
chr4:111,224,155–111,224,162	A-rich	SscSINEA8	Forward	*ENSSSCG00000006842*	ENSSSCT00000007493.5	Middle
chr4:89,507,153–89,507,167	A-rich	SscSINEA4	Reverse	*ENSSSCG00000062362*	ENSSSCT00000099995.1	Second-to-last
chr4:89,536,268–89,536,290	A-rich	SscSINEA4	Reverse	*ENSSSCG00000059629*	ENSSSCT00000103484.1	Last one
chr4:89,568,318–89,568,346	A-rich	SscSINEA4	Reverse	*ENSSSCG00000055244*	ENSSSCT00000097194.1	Second-to-last
chr5:15,123,190–15,123,212	A-rich	SscSINEB2	Forward	*LMBR1L*	ENSSSCT00000074724.2	Last one
chr5:18,076,941–18,076,954	A-rich	SscSINEA7	Reverse	*KRT3*	ENSSSCT00000104455.1	Last one
chr5:62,440,814–62,440,836	A-rich	SscSINEA2	Forward	*A2M*	ENSSSCT00000073778.2	First one
chr5:64,190,282–64,190,304	A-rich	SscSINEA2	Reverse	*TAPBPL*	ENSSSCT00000000767.4	Middle
chr5:79,572,418–79,572,440	A-rich	SscSINEA2	Reverse	*ENSSSCG00000046998*	ENSSSCT00000070014.2	First one
chr6:14,888,767–14,888,789	A-rich	SscSINEA4	Reverse	*ENSSSCG00000055830*	ENSSSCT00000098361.1	Last one
chr6:34,529,339–34,529,362	A-rich	SscSINEA8	Forward	*ADCY7*	ENSSSCT00000031919.4	Last one
chr6:57,439,997–57,440,006	A-rich	SscSINEC2	Forward	*ENSSSCG00000054543*	ENSSSCT00000100470.1	First one
chr7:101,631,283–101,631,315	(AAAAC)n	SscSINEA6	Reverse	*ENSSSCG00000044216*	ENSSSCT00000069832.1	Last one
chr7:41,507,618–41,507,624	A-rich	SscSINEA7	Forward	*PLA2G7*	ENSSSCT00000096961.1	First one
chr7:76,649,687–76,649,709	A-rich	SscSINEB1	Forward	*ENSSSCG00000054641*	ENSSSCT00000102255.1	Last one
chr8:71,573,309–71,573,323	A-rich	SscSINEC3	Forward	*PPEF2*	ENSSSCT00000075235.2	First one
chr9:66,523,062–66,523,119	(AAG)n	SscSINEA1	Reverse	*ENSSSCG00000021041*	ENSSSCT00000085293.2	Last one
chr10:19,985,460–19,985,482	A-rich	SscSINEA2	Reverse	*ASPM*	ENSSSCT00000097781.1	Middle
chr10:43,003,120–43,003,134	A-rich	SscSINEA7	Reverse	*PTCHD3*	ENSSSCT00000101644.1	Last one
chr10:65,173,953–65,173,970	A-rich	SscSINEA3	Reverse	*GDI2*	ENSSSCT00000077549.2	First one
chr10:6,729,528–6,729,550	A-rich	SscSINEA2	Reverse	*NET1*	ENSSSCT00000037985.3	Last one
chr11:25,450,394–25,450,478	AG-composite	SscSINEB1	Reverse	*VWA8*	ENSSSCT00000061114.3	Last one
chr11:44,897,857–44,897,877	A-rich	SscSINEA6	Reverse	*ENSSSCG00000009458*	ENSSSCT00000010368.3	Last one
chr12:21,606,460–21,606,509	A-rich	SscSINEA1	Forward	*KRT12*	ENSSSCT00000059521.1	Last one
chr12:38,018,891–38,018,912	A-rich	SscSINEA2	Reverse	*ZNHIT3*	ENSSSCT00000096703.1	Last one
chr12:3,805,497–3,805,515	A-rich	SscSINEA9	Reverse	*ENSSSCG00000041583*	ENSSSCT00000077609.1	First one
chr13:12,683,696–12,683,719	(AAAC)n	SscSINEA7	Reverse	*NGLY1*	ENSSSCT00000061148.2	First one
chr13:190,640,632–190,640,654	A-rich	SscSINEA2	Reverse	*ENSSSCG00000060713*	ENSSSCT00000103861.1	First one
chr13:31,603,362–31,603,420	A-rich	SscSINEA1	Reverse	*ARIH2*	ENSSSCT00000059716.2	First one
chr13:33,247,135–33,247,157	A-rich	SscSINEA4	Reverse	*DOCK3*	ENSSSCT00000041721.2	First one
chr13:54,339,602–54,339,629	(AAAAC)n	SscSINEA6	Forward	*PPP4R*	ENSSSCT00000056573.3	First one
chr13:67,585,979–67,586,004	(AC)n	SscSINEA4	Forward	*ATG7*	ENSSSCT00000042380.3	Middle
chr15:75,895,526–75,895,545	A-rich	SscSINEA7	Forward	*FASTKD1*	ENSSSCT00000025574.4	Last one
chr17:47,766,680–47,766,702	A-rich	SscSINEA4	Forward	*ENSSSCG00000035296*	ENSSSCT00000095053.1	First one
chr18:10,247,389–10,247,439	AG-composite	SscSINEA7	Forward	*ENSSSCG00000051926*	ENSSSCT00000102503.1	Last one
chr18:11,403,649–11,403,685	(AAC)n	SscSINEA1	Reverse	*ENSSSCG00000056501*	ENSSSCT00000091959.1	Last one

* Based on the transcription direction of the protein-coding gene, the SINE tail indicates the location of the inserted EXON. “First one” represents the first EXON at the 5′ end of the transcription direction; “Last one” represents the last EXON at the 5′ end of the transcription direction; “Second-to-last” represents the second-to-last EXON at the 5′ end of the transcription direction; other insertion positions are indicated by “Middle”.

## Data Availability

All data are available in the main text and/or the Supplementary Materials.

## Supplementary Materials

The following supporting information can be downloaded at: https://www.mdpi.com/article/10.3390/genes17020200/s1. Figure S1: Based on the predefined cutoff sequences of the SINEA-C subfamily consensus sequence, the 5' end boundary region of the SINE tail is determined;; Figure S2: Temporal distribution of absolute SINEA/B/C tail abundances across evolutionary time; Figure S3: Temporal dynamics of relative SINE tail type proportions; Table S1: SINE subfamily consensus sequences and SINEtail-scan pipeline test data; Table S2: Quantity of SINE tails types, genome information and “Other” SINE tail type description; Table S3: Distribution of SINE tail types insertion age; Table S4: Distribution of SINE tail types in chromosome; Table S5: Distribution of SINE tail types across functional genomic regions; Table S6: VWA8 mRNA sequences in Duroc and Banna pig genomes; Table S7: VWA8 protein sequences in Duroc and Banna pig; Table S8: AlphaFold structure prediction for VWA8 protein in PDB format.
