# Supplementary material for: Classification of SINE Tails in the Porcine Genome and Its Potential Impact on VWA8 Gene
Source: Genes (Basel). 2026 Feb 7;17(2):200. doi: 10.3390/genes17020200 (PMC12941198; doi:10.3390/genes17020200)
Supplement: Supplementary file 1 [file genes-17-00200-s001.zip › Figure S1-S2.pdf]

Consensus	Head	Body	Tail-region
SINEA1	-----------------------/	............/---------------------	-GCGGCCCAAGAAATAGCAACAACAACAACAACAAAAAAGACAAAAAGACCAAAAAAAAAAAAAAAAAAA
SINEA2	-----------------------/	............/---------------------	-GCGGCCCAAGAAATAGCAAAAAGACAAAAAAAAAAAAAA
SINEA3	-----------------------/	............/---------------------	-GCGGCCCAAGAAATAGCAAAAAGACAAAAAAAAAAA
SINEA4	-----------------------/	............/---------------------	-GCGGCCCTAGAAAAGGCAAAAAGACAAAAAAAAAAAAAA
SINEA5	-----------------------/	............/---------------------	-GCGGCCCTAGAAAAGACAAAAAGACAAAAAAAAAAAAAA
SINEA6	-----------------------/	............/---------------------	-GCGGCCCTAAAAAGACAAAAAACAAAAAAAAAAAAA
SINEA7	-----------------------/	............/---------------------	-GCGGCCCTAAAAAGACAAAAAAAAAAAAAA
SINEA8	-----------------------/	............/---------------------	-GCGGCCCTAAAAAGACAAAAAAAAAAAAAA
SINEA9	-----------------------/	............/---------------------	-GCGGCCCTAAAAAGAAAAAAAAAAAAAAAA
SINEA10	-----------------------/	............/---------------------	-GCGGCCNTAAAAAGAAAAAAAAAAAAAAAAA
SINEA11	-----------------------/	............/---------------------	-GCGGCCCTAGAAAAGGCAAAAAAAAAAAAAA
SINEB1	-----------------------/	............/---------------------	-GCGGCCCTAAAAAAGGCAAAAAAAAAAAAAAAAAAAAAAA
SINEB2	-----------------------/	............/---------------------	-GCGGCCCTAAAAAAGGCAAAAAAANAAAAAAAAAAAAAA
SINEB3	-----------------------/	............/---------------------	-GCGGCCAAAAAAGAAAAAAAAAAAAAAAA
SINEB4	-----------------------/	............/---------------------	-GCGGCCAAAAAAAGAAAAAAAANAAAAAAAAAAAAAA
SINEB5	-----------------------/	............/---------------------	-GCGGCCAAAAAAAGAAAAAAAANAAAAAAAAAAAAAA
SINEB6	-----------------------/	............/---------------------	-GCGGCCGTAAAAAGAAAAAAAAAAAAAAATCGT
SINEC1	-----------------------/	............/---------------------	-GCATGGCCAAAAAAAAAA
SINEC2	-----------------------/	............/---------------------	-GCATGGCCAAAAAAAAA
SINEC3	-----------------------/	............/---------------------	-GCGCGGCCAAAAAAAAAAAAAAA
SINEC4	-----------------------/	............/---------------------	-GCGCGGCCAAAAAAAAAAAAA
SINEC5	-----------------------/	............/---------------------	-GCGCGGCCAAAAAA
SINEC6	-----------------------/	............/---------------------	-GCGTGGCCAAAAAAAAAAAAAAAAAAAAA
SINEC7	-----------------------/	............/---------------------	-GCGTGGCCAAAAACAAACAAACAAACAAAAAA
SINEC8	-----------------------/	............/---------------------	-TGTGTGGCAAAAAAGAAAAAAAAAAAAAAATACAAAAAAAA

Figure S1. Based on the predefined cutoff sequences of the SINEA-C subfamily consensus sequence, the 5' end boundary region of the SINE tail is determined. The underlined part indicates the cutoff sequence of the corresponding SINE family.

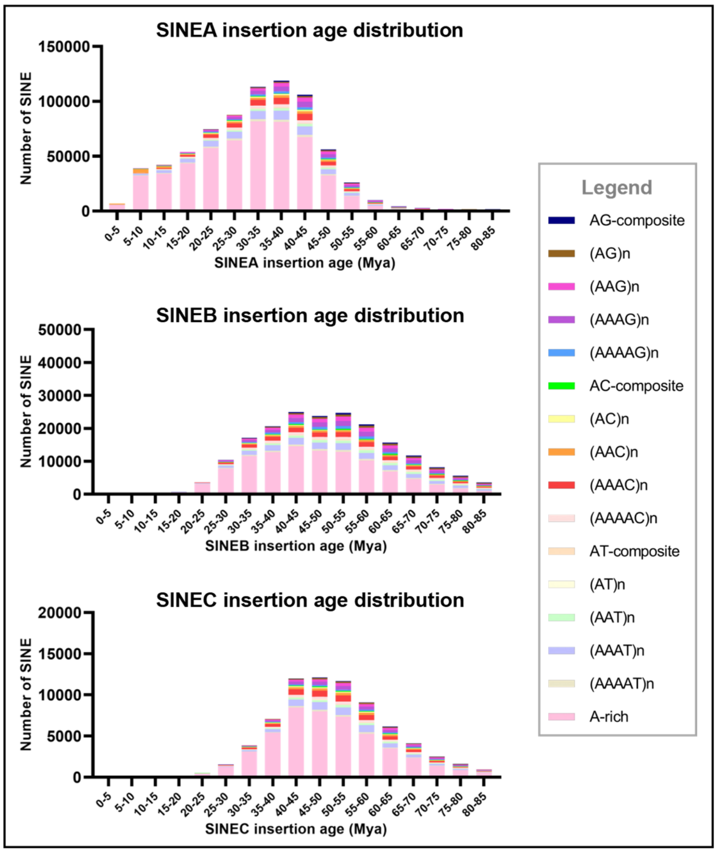

Figure S2. Temporal distribution of absolute SINEA/B/C tail abundances across evolutionary time. Stacked bar chart displaying the absolute number of SINE elements categorized by 16 tail structure types across 5-million-year (Mya) time intervals spanning 0-85 Mya. The x-axis represents SINE insertion age bins, while the y-axis quantifies the total number of SINE elements within each age interval.

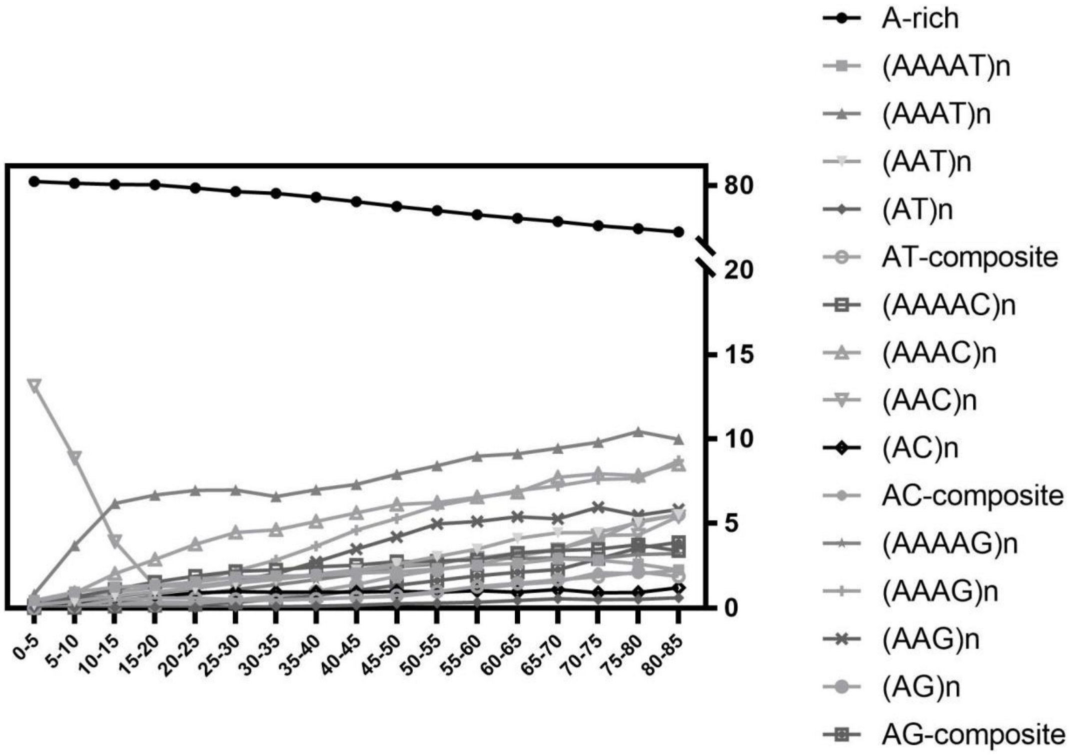

Figure S3. Temporal dynamics of relative SINE tail type proportions. Percentage line chart illustrating the relative compositional changes of 16 tail structure types across the same 5-Mya time intervals (0-85 Mya). The x-axis represents SINE insertion age point, while the y-axis displays percentage composition normalized to 100% within each temporal point.
